# Microbiological treatment failure associated with macrolide-resistant *Bordetella pertussis*

**DOI:** 10.1128/asmcr.00093-24

**Published:** 2025-03-11

**Authors:** David Prabhakar, Winkie Fong, Eby M. Sim, Annaleise R. Howard-Jones, Trang Nguyen, Suzanne Dempsey, Rebecca J. Rockett, Jen Kok, Sharon C.-A. Chen, Alexander C. Outhred, Vitali Sintchenko

**Affiliations:** 1 Microbiology and Infectious Diseases, Children's Hospital at Westmead 8538 , Sydney, New South Wales, Australia; 2 Center for Infectious Diseases and Microbiology-Public Health, Westmead Hospital, Western Sydney Local Health District 631938 , Sydney, New South Wales, Australia; 3 School of Medical Sciences and Sydney Infectious Diseases Institute (Sydney ID), Faculty of Medicine and Health, The University of Sydney 216920 , Sydney, New South Wales, Australia; 4 Center for Infectious Diseases and Microbiology Laboratory Services, Institute of Clinical Pathology and Medical Research, NSW Health Pathology 441551 , Sydney, New South Wales, Australia; Pattern Bioscience, Austin, Texas, USA

**Keywords:** pertussis, *Bordetella pertussis*, macrolide resistance, treatment outcomes, genome sequencing

## Abstract

**Background:**

Despite successful vaccination programs, many countries are experiencing a marked rise in pertussis cases since 2024, compounded by the emergence of macrolide-resistant *Bordetella pertussis* (MRBP). Here, we report the clinical, laboratory, and genomic features of MRBP isolated from a child with severe pertussis.

**Case Summary:**

An 11-week-old term infant with pertussis who required intensive care unit support remained *B. pertussis* culture-positive despite azithromycin therapy. Pre- and post-treatment isolates demonstrated phenotypic drug resistance and carried known 23S rRNA macrolide-resistance conferring mutations.

**Conclusion:**

The international spread of macrolide-resistant *B. pertussis* warrants the introduction of susceptibility testing for this pathogen.

## INTRODUCTION

*Bordetella pertussis* infection typically results in coughing paroxysms, but life-threatening pneumonia with pulmonary hypertension or respiratory arrest is a severe manifestation that can occur in infants (1). Despite successful vaccination programs, *B. pertussis* continues to circulate, and many countries, including Australia, experienced a marked rise in pertussis cases in 2024. This upsurge has been characterized by an age shift to older children and adults, vaccine escape, and emerging drug resistance (2). Erythromycin resistance in *B. pertussis* was first reported in 1993 (3) and was attributed to the A2047G mutation in 23S rRNA (4). Recently, cases of macrolide-resistant *B. pertussis* (MRBP) have been increasingly diagnosed worldwide (5–7), with the highest prevalence in China (8) and Vietnam (9). Here, we report the clinical, laboratory, and genomic features of MRBP isolated from an infant with severe pertussis who experienced microbiologic failure of azithromycin therapy. This is the first case of MRBP disease diagnosed in Australia.

## CASE PRESENTATION

An 11-week-old term infant became unwell on 2 August 2024 with cough, post-tussive vomits, and coryza. Prolonged coughing paroxysms, apnea, and central cyanosis without loss of consciousness prompted an emergency department presentation via ambulance. Three weeks prior, the patient and two siblings reported a mild, laboratory-confirmed respiratory syncytial virus (RSV)-B upper respiratory tract infection, which she fully recovered from. The patient was previously well and received her first dose of *B. pertussis* vaccine at 6 weeks. Her mother received acellular pertussis vaccine (Boostrix; GlaxoSmithKline) 15 days prior to delivery. No recent overseas travel for the child or family members was reported.

In the emergency department, the patient had two further apneic episodes and required intensive care unit admission for respiratory pressure support (bubble continuous positive airway pressure). She was commenced on intravenous (IV) azithromycin (10 mg/kg/day), cefotaxime (50 mg/kg/dose 6 hourly), and aciclovir (20 mg/kg/dose 8 hourly). Aciclovir was ceased after the second dose, and cefotaxime was ceased upon confirmation that her blood cultures remained negative after 48 hours of incubation. Azithromycin was switched to oral suspension (10 mg/kg/day) after the initial IV dose and given for five further doses (six daily doses in total). Household contacts were also provided with a course of azithromycin for prophylaxis via their general practitioner, though this was incomplete (parents received 3 days, siblings received 4 days; recommendation is 5 days).

A nasopharyngeal aspirate was collected from the patient during admission to the pediatric intensive care unit. Multiplex PCR (Seegene Allplex Respiratory Panel 4) detected *B. pertussis* (cycle threshold value [Ct] = 15), rhinovirus (Ct = 15), and, again, RSV-B (Ct = 25). *B. pertussis* colonies grew on Oxoid charcoal agar with horse blood with cephalexin (4 mg/L) after 48 hours of incubation. For this isolate CIDM-MRBP01, MIC for erythromycin and azithromycin by *E*-test (bioMérieux, France; performed on the same home-made charcoal blood agar without cephalexin) was assessed as >256 and 32 mg/L, respectively. The child was discharged after 8 days.

**Fig 1:**
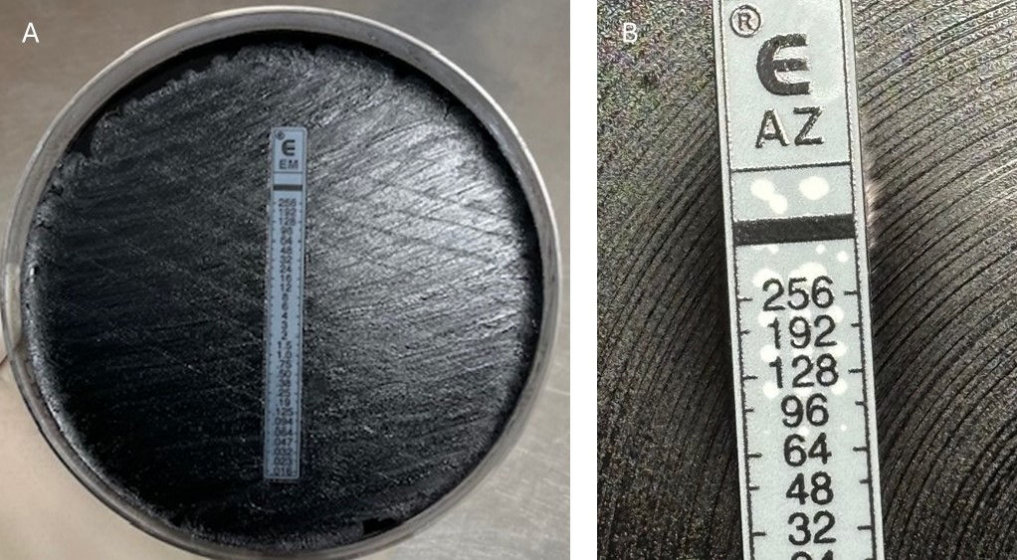
*E-*test of *B. pertussis* from this case on Oxoid charcoal agar with inoculation density equivalent of 0.5 McFarland. Bacterial growth in touch with the test strip of erythromycin (**A**) and azithromycin (**B**).

Following the detection of suspected MRBP in our patient, an outpatient follow-up was arranged on day 14 of illness (3 days post-discharge). At this time, the patient was still experiencing tussive episodes lasting approximately 30 seconds, without cyanosis, apnea, or vomiting. A 7-day course of trimethoprim with sulfamethoxazole (4 + 20 mg/kg/dose twice daily) was prescribed for the patient and her household contacts. A repeat nasopharyngeal swab was positive by multiplex PCR for rhinovirus (Ct = 25) and *B. pertussis* (Ct = 24), and *B. pertussis* was isolated again after 4 days of incubation. This post-treatment isolate CIDM-MRBP02 had *E*-test MIC > 256 mg/L for both erythromycin and azithromycin (Fig. 1). MIC for cotrimoxazole (0.032 mg/L) and ceftriaxone (0.032 mg/L) were wild type and consistent with susceptibility. *B. pertussis* and RSV-B were detected by multiplex PCR in a nasopharyngeal swab from the patient’s mother (Ct values of 40 and 37, respectively), but *B. pertussis* culture was negative. The patient was reviewed again one month later and had almost completely recovered, with only a mild dry nocturnal cough persisting. The child was readmitted with mild human metapneumovirus-associated bronchiolitis 2 months after initial presentation, and *B. pertussis* DNA was not detected on a nasopharyngeal aspirate at that time.

Genomic DNA from *B. pertussis* isolates from the case was extracted using the DNeasy UltraClean Kit (QIAGEN) with modifications (10). Short-read sequencing libraries were prepared using a Nextera DNA Prep Kit (Illumina) and sequenced on the NextSeq 2000 (Illumina). Long-read libraries were prepared from the same DNA extract using the SQK-RBK004 rapid barcoding kit (Oxford Nanopore Technologies, ONT). Sequencing was performed on MinION Mk1B (ONT). Short reads were quality checked and trimmed using FastQC v.0.11.3 and Trimmomatic v.1.0.4 (11). Long reads were filtered using Filtlong v.0.2.1 and assessed for contamination using Centrifuge v.1.0.4 (12). Hybrid assembly was performed using Dragonflye v.1.2.1 with Flye v.2.9.5-b1801 (13). One round each of long- and short-read polishing by Medaka v.1.11.3 employed the model “r941_e81_hac_g514” and Polypolish v.0.6.0 (14), respectively. Pertinent virulence factors were identified using BLASTN against an in-house *B. pertussis* database. The hybrid assemblies were annotated using Prokka v.1.14.6 (15). All genomic comparisons were performed against the reference genome *B. pertussis* strain Tahoma I (RefSeq accession: NC_002929.2; Table 1). Inferences of small genomic variation between the two genomes (Table 2) were made using Snippy v.4.6.0.

**TABLE 1:** Genomic coordinates of the pertactin coding sequence and the 23S rRNA

Coding sequence/rRNA	Tahoma I	CIDM-MRBP01	CIDM-MRBP02
Pertactin start	1098091	1413059	1413056
Pertactin end	1100823	1416855	1416852
23S first-copy start	2151159	2314483	2314407
23S first-copy end	2154040	2317364	2317288
23S second-copy start	2442207	2741526	2741452
23S second-copy end	2445088	2744407	2744333
23S third-copy start	3233759	3553802	3553743
23S third-copy end	3236640	3556683	3556624

**TABLE 2:** Genomic variations between CIDM-MRBP01 and CIDM-MRBP02 with IS481-associated variation filtered out

		Sequence in:	
Position in CIDM-MRBP01	Type^*a*^	CIDM-MRBP01	CIDM-MRBP02
241255	del	GC	G
479065	ins	T	TG
484894	del	AG	A
925846	del	GC	G
1219036	del	GC	G
1493612	ins	A	AGGG
1943911	snp and del	GG	C
2064240	del	CCG	C
2707534	ins	G	GGC
3094893	del	GC	G
3094939	del	CG	C
3209344	ins	A	AG
3388710	ins	T	TC
3445689	del	AG	A
3460728	del	GCC	G
3552408	ins	T	TCC
3589844	del	TG	T
3992387	ins	T	TG

^*a*^ ins: insertion; del: deletion; snp: single nucleotide polymorphism.

**Fig 2:**
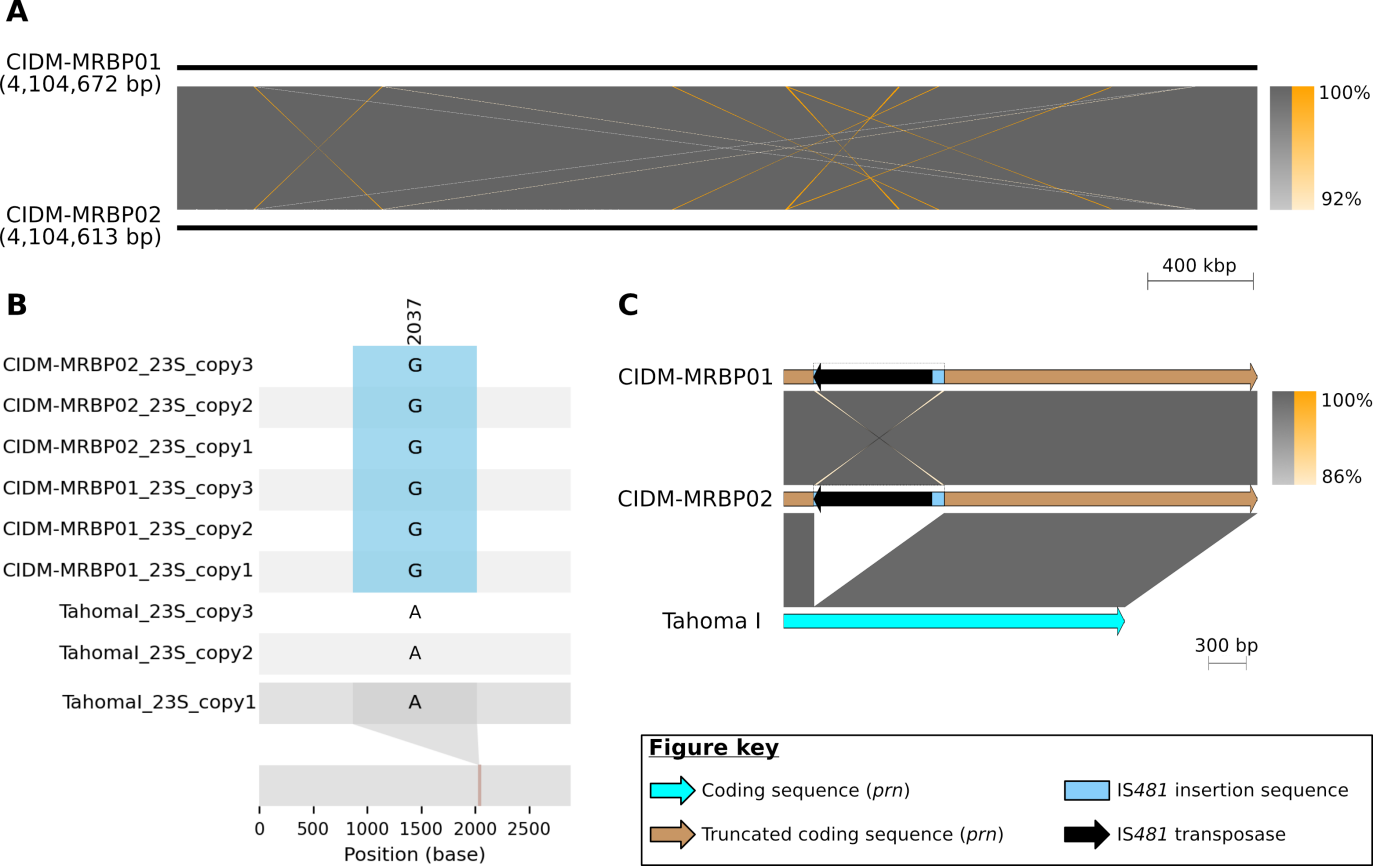
Genomic comparisons of the macrolide-resistant *B. pertussis* isolates CIDM-MRBP01 (pre-treatment with azithromycin) and CIDM-MRBP02 (post-treatment). (**A**) Pairwise comparison between genomes of CIDM-MRBP01 (Top) and CIDM-MRBP02 (Bottom). Gray shading indicates BLASTN matches (minimum length of 1,072 bp to filter out repetitive matches to all instances of IS*481* within the genome) on the same strand, and orange shading indicates matches to the opposite strand. The scale bar represents genome length, while the color gradient represents nucleotide identity. (**B**) Graphical representation of the A2047G mutation in both genomes. The 23S rRNA sequences from the publicly available genome *B. pertussis* Tahoma I (RefSeq accession: NC_002929.2) were used as reference. The positional discrepancy of this mutation, between positions 2,037 and 2,047, has been previously discussed (6). (**C**) Pairwise comparison of the pertactin coding sequences. Genome length and BLASTN identity are scaled according to the scale bar and color gradient, respectively. Panels A and C were generated using EasyFig v.2.2.4 (16). Panel B was generated using Snipit version 1.4 (17).

Both CIDM-MRBP01 (NCBI GenBank accession CP172429) and CIDM-MRBP02 (CP172575) genomes showed high levels of genomic similarity and synteny (Fig. 2). There were 18 instances of small genomic variations after filtering of indels within IS*481*, and both genomes were separated by one core single nucleotide polymorphism. Taken together, this suggested persistent infection rather than exogenous reinfection. All three copies of the 23S rRNA in each genome contained the A2047G mutation associated with macrolide resistance. Both genomes harbored the *ptxP3* allele as well as a pertactin gene (*prn*) disrupted by IS*481* insertion at nucleotide position 240 (Fig. 2). The virulence alleles were as follows: *ptxP3*/*ptxA*1/*prn2*(neg)/*fim3-1*/*fhaB1*.

## DISCUSSION

There were no reports of MRBP in Australia prior to this case. The patient had not traveled overseas and had no known contact with returned travelers; autochthonous acquisition is likely, implying that MRBP is already circulating in Australia. The virulence profile of the isolate represents a variant that is common in Asia. The disruption of the pertactin gene by insertion sequence IS*481* (Fig. 2C) could reduce the vaccine-induced protection in this case. Despite symptomatic improvement coinciding with azithromycin and cefotaxime therapy, microbiological failure was demonstrated by ongoing *B. pertussis* culture positivity, and the patient was re-treated with an alternative agent. Cotrimoxazole is currently used for the treatment or prophylaxis of pertussis in MRBP cases, though data on its clinical effectiveness are lacking. Other potential options include fluoroquinolones or beta-lactams (18). The therapeutic effect of 48 hours of cefotaxime remains uncertain in this case.

This documented microbiological failure of macrolide therapy demonstrates the importance of the timely recognition of MRBP in clinical settings to guide the optimal selection of antimicrobials and reduce the infectiousness of cases (19). It focused attention on the importance of bacterial culture and susceptibility testing in the laboratory diagnosis of pertussis. *B. pertussis* can be cultured from left-over transport media in bacterial swabs that are tested positive by PCR. Such a reflex culture approach might be a pragmatic option for cases of protracted or severe disease, including those requiring intensive care. It would support the use of respiratory swabs in universal transport media or nasopharyngeal aspirates for respiratory multiplex PCR testing. In recent decades, *B. pertussis* culture has been largely replaced by molecular testing as a more sensitive and rapid alternative and more suitable for automation. The emergence of MRBP has the potential to reverse this historical trend.

The use of nucleic acid amplification tests, which target the 23S rRNA A2047G mutation (20), is an appealing option for pathology providers since at this stage, no other molecular mechanisms of increased MICs to macrolides have been reported. However, bacterial culture remains essential for unearthing emergent markers of macrolide resistance and deciphering alternative mechanisms. Mutations in all three copies of the 23S rRNA lead to high levels of resistance, while mutations in only one copy may be associated with heterogeneous resistance (4). In our case, both *B. pertussis* genomes harbored the A2047G mutation in all three copies of the 23S rRNA, which is usually associated with MRBP; hence, our genomic analysis could not explain the different azithromycin MICs between the two strains. It is plausible that there was an unknown mutation within the regulatory or promotor genes or a hetero-resistance within *B. pertussis* populations.

In this report, we have presented a case of severe pertussis in an infant who required intensive care and remained culture-positive following azithromycin therapy. The microbiological investigation and genome sequencing have confirmed the presence of genomically closely related *B. pertussis* strains with A2047G mutation in all three copies of the 23S rRNA, which is associated with macrolide resistance in *B. pertussis*. The survival of implicated *B. pertussis* in respiratory secretions after azithromycin therapy and the increasing recognition of MRBP raises concerns about the potential public health impact of inadequate therapy during pertussis epidemics and the clinical risk to young infants in particular. This case highlighted the re-emerging role of routine culture for *B. pertussis* supported by susceptibility testing and detection of macrolide-resistance conferring mutations. Whether a test-of-cure for severe pertussis is warranted can be debated, but macrolide-resistant pertussis deserves further monitoring and necessitates a re-evaluation of current microbiology testing practices.
